# Metal ions leachables from fake orthodontic braces incubated in simulated body fluid

**DOI:** 10.1186/s12903-021-01880-x

**Published:** 2021-10-08

**Authors:** Riyam Haleem, Noor Ayuni Ahmad Shafiai, Siti Noor Fazliah Mohd Noor

**Affiliations:** Cluster of Craniofacial and Biomaterial Sciences, Advanced Medical and Dental Institute (IPPT), Universiti Sains Malaysia, 13200 Kepala Batas, Pulau Pinang, Malaysia

**Keywords:** Composition, Fake braces, Metal ion release, Orthodontic

## Abstract

**Background:**

The demand for fake braces usage in Southeast Asia are increasing but lack of certification and information on fake braces as medical devices from regulated bodies raised a concern towards its safety. The aim of this study was to determine the types of metal ion leachable from removable fake braces based on heavy metal ions present in metallic materials, immersed in simulated body fluid (SBF) and analysed using inductively coupled plasma atomic emission spectroscopy.

**Methods:**

Three sets of fake braces and one control were dissembled to only their brackets and archwires and immersed separately in SBF. They were placed in an incubator shaker at a temperature of 37 °C at 50 rpm. A 3.0 ml measurement of SBF was taken out from the sample containers at days 7, 14 and 28 and kept at − 20 °C for further analysis. Data were analysed using SPSS version 26.0 (IBM, Armonk, USA) (*P* < 0.05). Descriptive and one-way ANOVA analyses with *Bonferroni* post hoc tests were used to assess the significant differences between the metal ions released in SBF from the control samples and fake braces.

**Results:**

All 23 elements under investigation except Si ions were detected from the control samples and fake braces. There were significant increased K ions and reduced levels of Mg ions from the fake archwires and brackets. Most ions released were less than 10 mg/L (Ti, V, Cr, Mn, Fe, Co, Ni, Cu, Zn, Mo, Cd, Pb, Al) or 1 mg/L (Li, Ba) into the SBF medium.

**Conclusion:**

There were significant release of Ca and K ions from the fake samples. Elements such as Li, Ba, Ti, V, Cr, Mn, Fe, Co, Ni, Cu, Zn, Mo, Cd and Sb had increased in concentration at day 7 and the concentration plateaued until day 28.

## Background

The use of fake orthodontic braces in Southeast Asia is a trend among teenagers, who see them as stylish accessories and alternatives for those who cannot afford proper orthodontic treatment at a dental clinic. Furthermore, the long waiting lists for orthodontic treatment at government dental clinics, which is limited to subjects who have dental malocclusion with Index of Orthodontic Treatment Need (IOTN) scores of 4 and 5, may have increased the use of fake braces [1]. Subjects having IOTN scores of less than 4 would normally seek treatment in private clinics, which tend to be costly compared to the prices at government dental clinics.

High costs and delays in receiving orthodontic treatment are two of the many reasons why subjects opt for illegal orthodontic practice, which not only offers cheaper alternatives, but also easier access as the fixation of braces is done at home or in salons [2]. Subjects are not required to attend multiple visits for dental examinations and reviews after the fixation of the appliances. These practices are sometimes unsupervised by guardians or parents, while these offers seem appealing to teenagers and subjects with financial constraints, hence the increase in the demand for fake braces. Fake braces are commonly available as fixed and removable types. The fixed ones are glued to the teeth, while the removable ones (fashion braces) are attached to the teeth by hooks and worn as a temporary measure. These fake braces are sold extensively on online platforms.

Fake braces usage poses risks to subjects since the source and composition of the materials are unknown [3]. The packaging does not provide information of the manufacturer, date of manufacturing or expiry date, hence the query whether the materials are even certified from regulated bodies. Few studies are available regarding the toxic effects of fake braces on humans. These are either under-reported or difficult to document cases related to fake braces, since most wearers rarely disclose the use of such braces to friends or peers. Many in vitro and laboratory studies have been conducted on as-received brackets, that is, brackets from manufacturers commonly used in dental clinics [4, 5], but rarely have such studies been performed on fake braces [6]. Hence, there is a need to study the types of metal ion leachable from fake braces available on the Malaysian market. Therefore, the aim of this study was to determine the metal ion leachable from removable fake braces or so-called fashion braces, that were immersed in simulated body fluid (SBF) and analysed using inductively coupled plasma atomic emission spectroscopy (ICP-OES).

## Methods

### Sample preparation

**Fig. 1:**
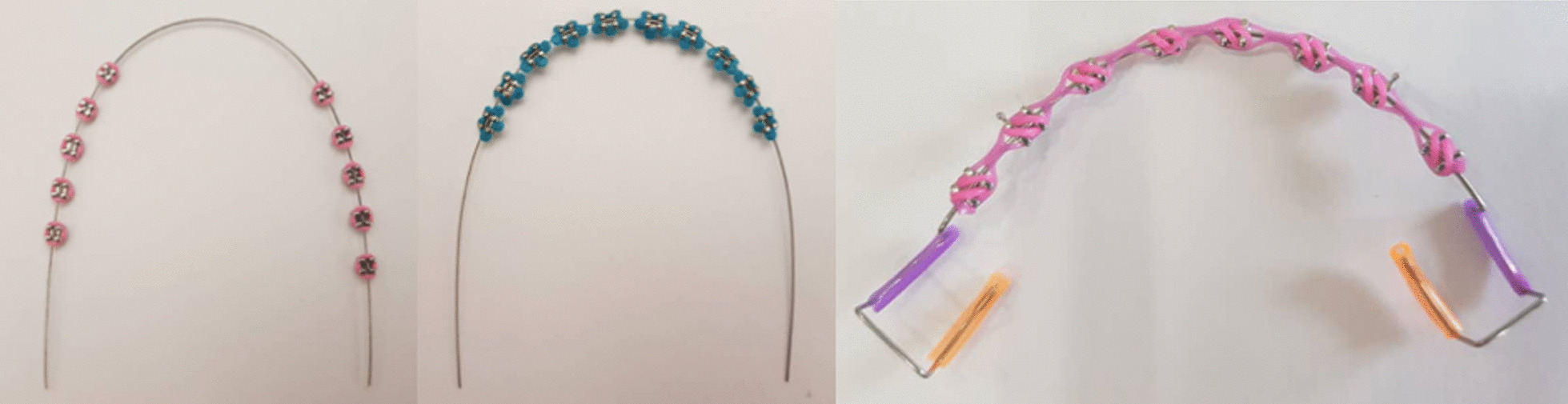
Types of fake braces

Three sets of fake braces (fashion braces) were bought online (www.shopee.com.my) (Fig. 1). These fake braces were searched using the word ‘fake braces’, and product marketed as ‘click braces’ or ‘click power chain’; with the highest amount of purchase and different name of suppliers were chosen since highest purchases indicated high sales due to either cheap prices, increased demand from users or popular usage among wearers. The appliances were then dissembled, whereby in each type of fake braces, four brackets (B) and four archwires (AW) were cut into lengths of 10 mm each. The standard sample was represented by a 0.018″ stainless steel archwire (3 M Unitek) and an orthodontic metal bracket (mini MBT 022 Hook 345). Each sample was weighted prior to soaking in SBF (Table 1).

**Table 1:** The weight of samples in milligram (mg)

	Control Mean (SD)	Type 1 Mean (SD)	Type 2 Mean (SD)	Type 3 Mean (SD)	*P* value^a^
Bracket	67.8 (0.59)	61.3 (1.06)	53.0 (1.03)	52.8 (0.95)	0.09
Archwire	13.4 (0.09)	13.5 (0.24)	23.4 (0.33)	6.8 (0.08)	0.82

^a^One sample *t* test

### SBF preparation

The SBF medium was prepared, according to Kokubo and Takadama [7], at pH 7.4 and the composition is shown in Table 2. The amount of SBF required for each sample was calculated according to the weight of the samples. The concentrations of SBF were 0.5 mg/ml for the archwire and 2 mg/ml for the bracket. The wires and brackets were immersed separately in SBF, and placed in an incubator shaker (IKa Ks 4000, USA) at a temperature of 37 °C at 50 rpm to mimic the body temperature and salivary flow. At designated time points (days 7, 14 and 28), 3.0 ml of SBF was taken out from the sample containers and kept at − 20 °C in a freezer.

**Table 2:** The weight and volume of reagents for 1000 ml simulated body fluid (SBF)

Reagents	Amount
NaCl	8.035 g
NaHCO_3_	0.355 g
KCl	0.225 g
K_2_HPO_4_·2H_2_O	0.231 g
MgCl·6H_2_·O	0.311 g
1.0_M_ HCl	39 ml
CaCl_2_·2H_2_·O	0.292 g
Na_2_SO_2_	0.072 g
Tris 1 T	6.118 g
1.0_M_ HCl	0–5 ml

### Elemental analysis

On the day of analysis, samples were thawed to room temperature, filtered using a 0.22 μm syringe filter and diluted by a factor of 10 in deionised water. The elemental analyses of the control and fake braces were performed using ICP-OES (Optima 8000, Perkin Elmer, USA). A standard calibration curve for each ion was obtained by preparing a standard solution containing 23 elements at 100 ppm (Sigma-Aldrich Multielement Standard Solution 6 for ICP) according to manufacturer’s instructions (Table 3). This was to ensure that the concentrations of metallic ions released were within the instrument’s range. Each sample was measured in triplicate.

**Table 3:** List of elements with its absorption wavelength used in ICP-OES

Element	Wavelength (nm)
Calcium (Ca)	422.673
Cobalt (Co)	238.892
Cadmium (Cd)	228.802
Iron (Fe)	259.939
Nickel (Ni)	231.604
Lead (Pb)	283.306
Zinc (Zn)	213.857
Chromium (Cr)	357.869
Magnesium (Mg)	285.213
Copper (Cu)	324.752
Vanadium (V)	311.071
Titanium(Ti)	368.519
Antimony (Sb)	217.582
Molybdenum (Mo)	281.616
Sodium (Na)	589.592
Lithium (Li)	670.784
Aluminium (Al)	309.271
Boron (B)	182.578
Barium (Ba)	455.403
Potassium (K)	766.490
Manganese (Mn)	257.610
Phosphorus (P)	214.914
Silicon (Si)	251.611

### Statistical analysis

Data were compiled on an Excel sheet and analysed using SPSS version 26.0 (IBM Inc, Armonk, NY, USA) with a significance level of 5%. Descriptive and one-way ANOVA analyses with post hoc tests using *Bonferroni* were used to ascertain the significance of differences between the metal ions released in the SBF from the control samples and fake braces.

## Results

In general, all the elements under investigation, except silicon (Si) ions, were detected from the control samples and fake braces. Sodium (Na), potassium (K) and calcium (Ca) ions revealed the highest levels of released ions from the fake braces, with concentrations of more than 100 mg/L during the 28-day period of immersion. The mean concentration values of these ions, with the significant differences from the control, are shown in Tables 4, 5 and 6. There were significant increases of K ions and reduced levels of magnesium (Mg) ions from fake archwires and brackets throughout the immersion period, in comparison to the control samples.

**Table 4:** Ca ion release (mg/L) during 28 days immersion period presented as Mean (SD)

	Control	Type 1	Type 2	Type 3	*F* statistic (*df*)	*P* value^a^
Bracket (B)						
Day 7	146.84 (0.83)	139.73 (1.74)	180.85* (5.62)	159.5* (0.31)	110.44 (3,8)	0.00
Day 14	147.81 (1.13)	165.60* (8.05)	138.60 (0.27)	158.80 (0.53)	25.66 (3,8)	0.00
Day 28	93.50 (0.43)	101.71* (1.14)	143.71* (1.55)	142.77* (1.74)	1222.45 (3,8)	0.00
Archwire (AW)						
Day 7	108.33 (0.97)	147.51* (2.30)	132.46* (2.50)	161.90* (0.75)	482.40 (3,8)	0.00
Day 14	120.80 (3.52)	160.90* (1.85)	124.97 (0.74)	155.25* (2.18)	239.10 (3,8)	0.00
Day 28	93.16 (1.64)	154.00* (1.53)	142.42* (1.27)	150.03* (0.50)	1389.42 (3,8)	0.00

^a^One-way ANOVA

*Statistically significance difference between control and fake sample using post hoc test *Bonferroni* *P* < 0.05

**Table 5:** Na ion release (mg/L) during 28 days immersion period presented as Mean (SD)

	Control	Type 1	Type 2	Type 3	*F* statistic (*df*)	*P* value^a^
Bracket (B)						
Day 7	40.62 (24.98)	81.71 (55.04)	106.90 (0.17)	100.19 (0.55)	2.92 (3,8)	0.10
Day 14	56.53 (31.45)	98.39 (10.61)	110.70* (0.68)	102.27 (7.48)	6.06 (3,8)	0.02
Day 28	282.43 (218.15)	109.65 (0.54)	101.44 (0.17)	103.99 (5.66)	1.99 (3,8)	0.20
Archwire (AW)						
Day 7	227.69 (137.57)	102.48 (0.93)	98.72 (13.24)	109.49 (0.42)	2.43 (3,8)	0.14
Day 14	164.56 (123.29)	105.15 (0.01)	134.73 (5.51)	106.77 (0.44)	0.62 (3,8)	0.62
Day 28	176.74 (123.24)	114.46 (0.01)	100.51 (2.28)	102.70 (0.84)	1.02 (3,8)	0.43

^a^One-way ANOVA

*Statistically significance difference between control and fake sample using post hoc test *Bonferroni* *P* < 0.05

**Table 6:** K ion release (mg/L) during 28 days immersion period presented as Mean (SD)

	Control	Type 1	Type 2	Type 3	*F* statistic (*df*)	*P* value^a^
Bracket (B)						
Day 7	11.95 (0.01)	218.35* (0.35)	172.35* (0.45)	180.25* (3.70)	7157.99 (3,8)	0.00
Day 14	11.91 (0.01)	171.70* (0.38)	184.35* (3.10)	160.50* (2.74)	4535.66 (3,8)	0.00
Day 28	11.91 (0.01)	165.00* (1.17)	188.85* (0.65)	147.43* (0.72)	32,649.95 (3,8)	0.00
Archwire (AW)						
Day 7	11.89 (0.01)	190.55* (1.50)	166.70* (2.56)	202.90* (0.94)	9826.35 (3,8)	0.00
Day 14	12.00* (0.31)	196.25* (1.91)	196.45* (4.14)	173.25* (2.78)	3334.76 (3,8)	0.00
Day 28	13.36* (0.26)	204.30* (1.58)	174.55* (0.31)	187.00* (1.52)	19,198.11 (3,8)	0.00

^a^One-way ANOVA

^*^Statistically significance difference between control and fake sample using post hoc test *Bonferroni* *P* < 0.05

Transition metals, which include elements like titanium (Ti), vanadium (V), chromium (Cr), manganese (Mn), iron (Fe), cobalt (Co), nickel (Ni), copper (Cu), zinc (Zn), molybdenum (Mo), and cadmium (Cd), showed similar patterns in terms of the ions released from both the control and fake archwire and bracket samples. Levels of these transition metal ions increased from day 0 to day 7, then plateaued until day 28. The concentrations of these ions that leached out from the fake braces were all below 10 mg/L. The only element which showed a significant difference to the control was Mo at day 7 (AW3), day 14 (AW1 and AW2) and day 28 (all types of fake archwires).

Post-transition metals like lead (Pb) and aluminium (Al) were also released at levels of no more than 10 mg/L in the immersion medium. Pb was significantly increased in comparison to the control at day 14 (AW2) and 28 (AW3). B1 showed significant increases in Pb ions at days 7 and 28. Al significantly increased at days 7, 14 and 28. Phosphorus (P) ions, on the other hand, were significantly elevated on all days of immersion for both fake archwires and brackets.

**Fig. 2:**
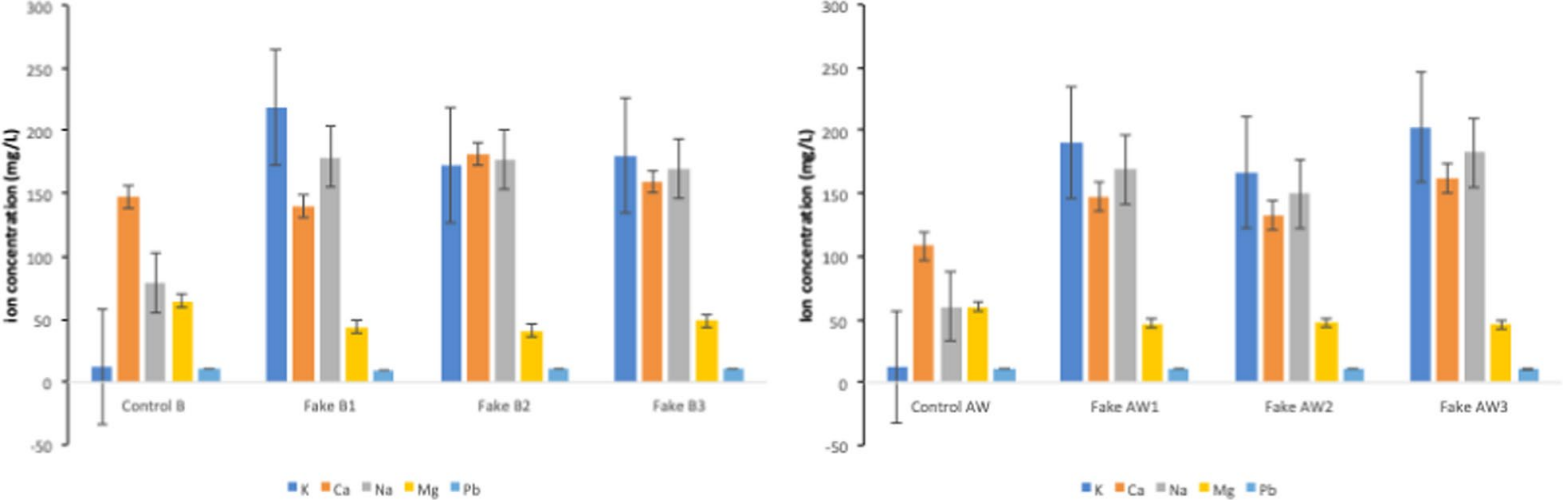
Top five highest ion released from each type of fake braces and control at Day 7

Lithium (Li) and barium (Ba) were released at low values, less than 1.0 mg/L, with no significant difference to the control samples. The release of antimony (Sb) ions in the fake samples showed no significant changes from the control. Boron (B) was only detected at day 28 for the fake archwires, while B had significantly increased for all fake brackets at days 7 and 14, as had B1 and B2 at day 28. The top five highest levels of ions released from each type of fake braces and the control are shown in Fig. 2.

## Discussion

To date, there have been two studies on fake brackets composition. Nasir et al. [6] examined several as-received fake brackets using a scanning electron microscope (SEM) equipped with an energy-dispersive spectroscopic (EDX) detector. It was concluded that the bracket surfaces were distinctly inferior to those of the standard brackets and no heavy metals such as Pb, mercury (Hg) and arsenic (As) were present [6]. Another paper by the same author reported high releases of Cr, Mn and Ni ions from fake orthodontic brackets immersed in artificial saliva [8].

SBF is commonly used in research as a standard medium with which to assess the bone-binding ability of dental implants by examining their ability to form an apatite layer. The apatite layer can be produced on the implant surface when immersed in SBF, as its ion concentration is close to human blood plasma [9]. For this reason, this study utilised SBF as the immersion medium and it could also be used as simulated media for determining the corrosion behaviour of biodegradable metallic materials [10]. Although blood is not in direct contact with fake braces on the teeth, it is possible that metal ions would leach into the blood vessels as a consequence of gingivitis due to the subject’s poor oral hygiene and when the duration of orthodontic treatment increases [11]. The best medium would be the saliva of patients wearing fake braces but it is unethical to recruit subjects to wear fake braces and it is difficult to find subjects who wear fake braces, as this is illegal.

Metal leaching from fake braces was compared with control samples since metal leachables from fake braces are unknown. Furthermore, the control samples that are commonly used in clinics are the best form of reference for the limit of safe ion release. Using eyeballing method, it was decided to use 0.018″ stainless steel archwire as the control archwire since the physical structure of the fake archwire used in this study was hard and round in diameter, similar to the shape of the control. Indeed, using stainless steel wire as control and comparison to the fake braces also support the notion that the fake braces price is low suggesting that it may originate from the common stainless steel or metallic based product.

The weight of the fake brackets and archwire samples and control has no significant differences indicating that they were comparable and the volume of SBF for sample immersion was set according to the weight of each sample to ensure consistency during experimental procedures. Instructions in the fake braces packaging also stated that they are temporary accessories and not meant to be fixed to the teeth, hence the choice of stainless-steel wire rather than the more resilient and springy nickel titanium (NiTi) archwire. NiTi archwire would be more suitable for use as a control in studies using fixed fake braces as this material presents a low modulus of elasticity and excellent springback, which is intended to help with the alignment of teeth [12].

In this study, the fake braces samples released Na, Ca, and K ions with levels of more than 100 mg/L over the 28-day period. K ion levels were significantly raised, about ten times higher in all types of fake braces from day 7 to day 28. Meanwhile, the control samples only released up to 13.26 mg/L of K ions at day 28. Nevertheless, the recommended daily intake for K in adult is much higher at 3.5 g/day by World Health Organisation and it is known that K ions are beneficial in promoting natriuresis and diuresis in the body. K ions release in these fake braces was considered safe but combination with other consumption of high K diet intake may produce significant increase of K ions resulting in hyperkalaemia. Patients at risk include those with chronic kidney disease, congestive heart failure and diabetes mellitus [13].

Ca ions are the most abundant in the body as they play vital roles in developing healthy bone, regulating muscle contraction, involvement in the blood clotting mechanism and also as co-factors for many enzymes [14]. The absorption of Ca into the body depends on other additional factors, such as enough vitamin D, vitamin C and some amino acids in the intestines. Therefore, in this study, the significantly higher levels of Ca ions released in almost all types of fake brackets and archwires, compared to the control samples, posed health concerns, except for B1 at day 7, and B2, B3 and AW2 at day 14. The recommended calcium intake is 800 mg per day [14], so it was suggested that the level of Ca ions in fake braces was still at a safe dose.

As highlighted, SBF is commonly used to investigate the bioactivity and corrosion resistance of materials, and it is also widely used for the biomimetic deposition of amorphous calcium phosphate (CaP) at 37 °C in biomedical materials. The reduced amounts of Ca and P ions released from day 7 to day 28 may indicate that some form of apatite had formed on the sample’s surfaces, so further investigations using, for example, FESEM and EDX, are needed to confirm its presence.

Interestingly, Na was found to be higher in the control samples. The highest release of Na ions was reported in the control bracket at 250 mg/L at day 28 and 154.05 mg/L from the control archwire at day 7 of immersion. Both the bracket and the control were made from stainless steel that roughly contained this composition percentage: Cr (18–20%), Ni (8–10.5%), C (0.08%), Mn (2.00%), Si (1.00%), P (0.045%), S (0.03%), N (0.1%), and Fe (balance); in this composition, no Na ions are incorporated. The only explanation for this could be the manufacturing process. Miyazaki et al. [15] reported NaOH and heat treatment during the manufacture of tantalum metal had accelerated apatite formation, which resulted in steep increases in Na concentration within day 1 of immersion in SBF. It was noted as a limitation of this study that more control samples from different manufacturers should be included.

The highest types of ions released from the control and fake braces samples were K, Ca, Na, Mg and Pb, which indicated that these ions were part of the composition. However, the percentage of ions release did not represent the proportional composition of the material [16] in which these percentages were not counted in the current study. However, based on the weight of the samples that were weighted before incubation in SBF and also the designated dose for archwires and brackets, the estimation of the ions released from each sample can be estimated if the actual composition of the materials is known especially during fabrication. Indeed, since the fake braces came without proper content or composition details, these calculations cannot be done accurately. Therefore, the precise elemental composition used in fake braces could not be determined based on this result.

Many studies on the release of metal ions have focused on transition metals such as Ti, Cr, Ni, Fe, Cu and Zn, as they make up the composition of orthodontic appliances. It is important to determine their biocompatibility and stability inside the oral cavity as they are marketed worldwide and used in patients over a long period of time. Ni and Cr are the most concerning of ions released, as they can initiate allergic reactions [17]. In this study, it was noted that releases of Ti, V, Cr, Mn, Fe, Co, Ni, Cu, Zn, and Cd ions were all below 10 mg/L over the 28-day immersion period, with no significant difference to the respective control samples, except for Mo. Nasir et al. [8] also found less than 10 mg/L of Ni, Cr and Mn were released but the differences were significant compared to the control at day 1 for Cr and Mn, and at day 14 for Ni. It can be suggested that a systemic toxic effect from these ions is unlikely for fake braces, but if these results were to be compared with the daily dose recommended by WHO [18], some of the elements were higher than their limit. Small quantities of metal ions in orthodontic appliances can cause allergic reactions [16], and fake braces that are worn in the oral cavity unsupervised for a long time can have detrimental effects on the body. If these appliances are in contact with the inflamed gingival, through which the released ions may continuously infuse into the blood capillaries, minute changes to the ion concentrations in the blood could also cause serious illness.

There are limitations to this study which includes only one control samples were used. It is suggested for at least two or three control samples to be constituted to increase the validity of the result in the future study. Only three samples of fake braces were chosen due to the constraint with the analysis cost; hence the results should be appraised with caution. The information of the manufacturer was not included in the packaging sold by the online seller whom most of the time are non-dental based seller (personal online seller) [3], hence there is possibility that these fake braces rooted from the same or different manufacturers sought by the online seller. Thus, making it difficult for the current research to determine exact material compositions. Nevertheless, the use of online platform for obtaining fake braces samples represents the purchase trends of these items by teenagers. It is worth noting that the incubation of samples in SBF cannot replicate the complex oral environment which has a continuous dynamic salivary flow. Some studies have practised introducing a new immersion medium to avoid the supersaturation of ions released [16] and the immersion medium in this study was used continuously throughout the 28 days. Continuous immersion, however, represents the cumulative level of ion release in the body. Within the limitation of this study, metallic ion leaching from fake braces needs to be investigated further in terms of the biocompatibility and the determination of doses that are toxic to human cells.

## Conclusion

Fake orthodontic braces released more than 100 mg/L of Na, Ca, and K ions during the immersion period; Ca and K levels were significantly different to the control samples.

Elements such as Li, Ba, Ti, V, Cr, Mn, Fe, Co, Ni, Cu, Zn, Mo, Cd and Sb had increased in concentration at day 7 and the concentration plateaued until day 28.

## Data Availability

The data underlying this article will be shared on reasonable request to the corresponding author.

## Abbreviations

ANOVA: Analysis of variance
SBF: Simulated body fluid
IOTN: Index of Orthodontic Treatment Need
